# Machine Learning–Based Comparative Analysis of Blood Cell–Derived Inflammatory Indices for Predicting MAFLD and Liver Fibrosis: Evidence From NHANES 2017–2020

**DOI:** 10.1002/jcla.70349

**Published:** 2026-09-15

**Authors:** Hao Chen, Qinmei Chen, Xiangqian Wang, Yixian He, Xiqiao Zhou

**Affiliations:** ^1^ Yancheng TCM Hospital Affiliated to Nanjing University of Chinese Medicine Yancheng Jiangsu China; ^2^ The First Clinical Medical College of Nanjing University of Chinese Medicine Nanjing Jiangsu China; ^3^ Department of Endocrinology Affiliated Hospital of Nanjing University of Chinese Medicine, Jiangsu Province Hospital of Chinese Medicine Nanjing Jiangsu China

**Keywords:** fatty liver disease, inflammatory indices, machine learning, MAFLD

## Abstract

**Background:**

Metabolic dysfunction associated fatty liver disease (MAFLD) is linked to chronic low‐grade inflammation, but the diagnostic value of blood cell–derived inflammatory indices remains unclear. This study assessed their associations and predictive ability for MAFLD and liver fibrosis in a nationally representative population.

**Methods:**

This cross‐sectional study included 3501 participants from NHANES 2017–2020. Multivariable logistic regression was used to evaluate associations between six inflammatory indices (AISI, NLR, LMR, PLR, SII and SIRI) and MAFLD with or without liver fibrosis. Restricted cubic spline analysis assessed dose–response relationships, while receiver operating characteristic (ROC) curves and random forest analysis evaluated diagnostic performance and variable importance.

**Results:**

Multivariable logistic regression analysis showed that higher values of ln (AISI) and ln (SIRI) were significantly associated with increased odds of MAFLD, whereas ln (SII), ln (LMR) and ln (NLR) were not significantly associated with MAFLD (*p* > 0.05). In contrast, ln (PLR) was inversely associated with MAFLD. Among participants with liver fibrosis (LF), higher values of ln (AISI), ln (NLR) and ln (SIRI) were positively associated with MAFLD, whereas ln (PLR) remained inversely associated. No significant associations were observed for ln (LMR) or ln (SII) with MAFLD in the presence of LF. Among the evaluated inflammatory indices, AISI demonstrated the highest discriminatory performance for MAFLD (AUC = 0.752), whereas PLR showed the best performance for predicting liver fibrosis. These findings were further supported by machine learning analysis.

**Conclusion:**

Inflammatory indices showed distinct associations with MAFLD and liver fibrosis. AISI was most strongly associated with MAFLD, whereas PLR showed the best performance for fibrosis assessment.

## Graphical abstract

This research assesses the correlations and diagnostic efficacy of blood cell‐derived inflammatory indicators in predicting metabolic dysfunction‐associated fatty liver disease (MAFLD) and liver fibrosis using NHANES 2017–2020 data. AISI had the highest predictive capability for MAFLD, while PLR served as the most efficacious indicator for fibrosis risk. These results underscore the promise of simple, economical inflammatory indicators for early detection and risk assessment in clinical practice.

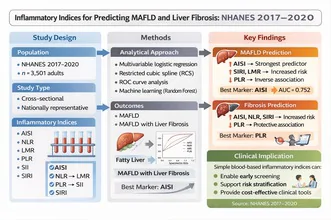

## 1 Introduction

Metabolically associated fatty liver disease (MAFLD), considered an alternative to the term nonalcoholic fatty liver disease (NAFLD) since 2020, is defined as hepatic steatosis (HS) with an association with metabolic dysfunction [1]. The diagnostic criteria for MAFLD include Type 2 diabetes mellitus (T2DM), metabolic dysfunction, and overweight/obesity. The criteria for diagnosing MAFLD point to the association between diabetes, CVD, and other metabolic factors [2], which are related to hepatic steatosis. With the increase in the prevalence of obesity globally, the prevalence of MAFLD is increasing, with 39% of the adult population affected [3]. MAFLD is a common chronic liver disease that is known for increasing the risk for cirrhosis, hepatocellular carcinoma, liver failure, and various extrahepatic complications such as T2DM, CVD, and chronic kidney disease [4]. The pathogenesis of MAFLD is multifactorial, with the “multiple‐hit” hypothesis being the most comprehensive theory so far [5]. This theory highlights the complex interaction between lipotoxicity, cytokine imbalance, inflammation, insulin resistance, innate immune system activation, oxidative stress, and gut microbiome dysbiosis [6]. Inflammation and immune system abnormalities are the major contributing factors to the progression from hepatic steatosis (HS) to liver fibrosis (LF) [7]. Inflammatory markers, which are simple, cost‐effective, and can be obtained from simple blood tests, have gained significant importance in the pathogenesis of metabolic and liver diseases [8].

Standard inflammation‐related indices include LMR, SII, PLR, NLR and SIRI. AISI has recently emerged as a novel composite inflammatory marker integrating neutrophils, monocytes, platelets and lymphocytes to better reflect systemic immune‐inflammatory status [9]. Recent studies have demonstrated the prognostic value of AISI in several chronic conditions, including cardiovascular disease, malignancies and metabolic disorders, where it has been associated with disease severity and adverse clinical outcomes [10]. However, evidence regarding its application in MAFLD remains scarce and its comparative diagnostic utility relative to other established inflammatory indices has not been fully elucidated. To our knowledge, few population‐based studies have comprehensively compared AISI with other inflammatory indices in MAFLD. Therefore, investigating the role of AISI alongside conventional inflammatory indices may provide additional insight into the inflammatory mechanisms underlying MAFLD and help identify more effective biomarkers for early detection and risk stratification.

This study utilized nationally representative NHANES 2017–2020 data to assess the associations between multiple inflammatory markers and MAFLD, as well as to compare their diagnostic performance. The findings aim to clarify inflammation‐related mechanisms in MAFLD and support the potential integration of these indices into early screening, risk stratification and targeted public health strategies.

This appears to be among the first nationally representative studies to use combined statistical methodology, including multivariate regression, restricted cubic spline modeling, ROC analysis and random forest classifier algorithms, to comprehensively compare different inflammatory markers derived from blood cell components and to analyze their utility for predicting the presence of MAFLD and liver fibrosis in an integrated fashion. Thus, the authors have developed a reliable framework for comparison of the inflammatory markers as potential clinical tests.

## 2 Materials and Methods

### 2.1 NHANES Study Population Selection (2017–2020)

NHANES, initiated in the 1960s and conducted continuously since 1999, employs a complex, stratified, multistage sampling methodology to ensure national representativeness of the U.S. population through standardized interviews, laboratory testing and physical examinations. For this study, NHANES 2017–2020 data comprising 15,560 participants were analyzed. As shown in Figure 1, exclusions included individuals aged < 18 years (*n* = 5867), missing data related to the definition of MAFLD (*n* = 5005), after the aforementioned criteria, 3501 remained for the final assessment. Participants in the included study (*n* = 3501) and excluded population (*n* = 9693) were compared using standardized mean difference (SMD). All SMD values were less than 0.1, indicating that the difference between the included and excluded participants was negligible. The National Center approved NHANES protocols for Health Statistics Ethics Review Board and Informed consent was provided by all participants.

**FIGURE 1:**
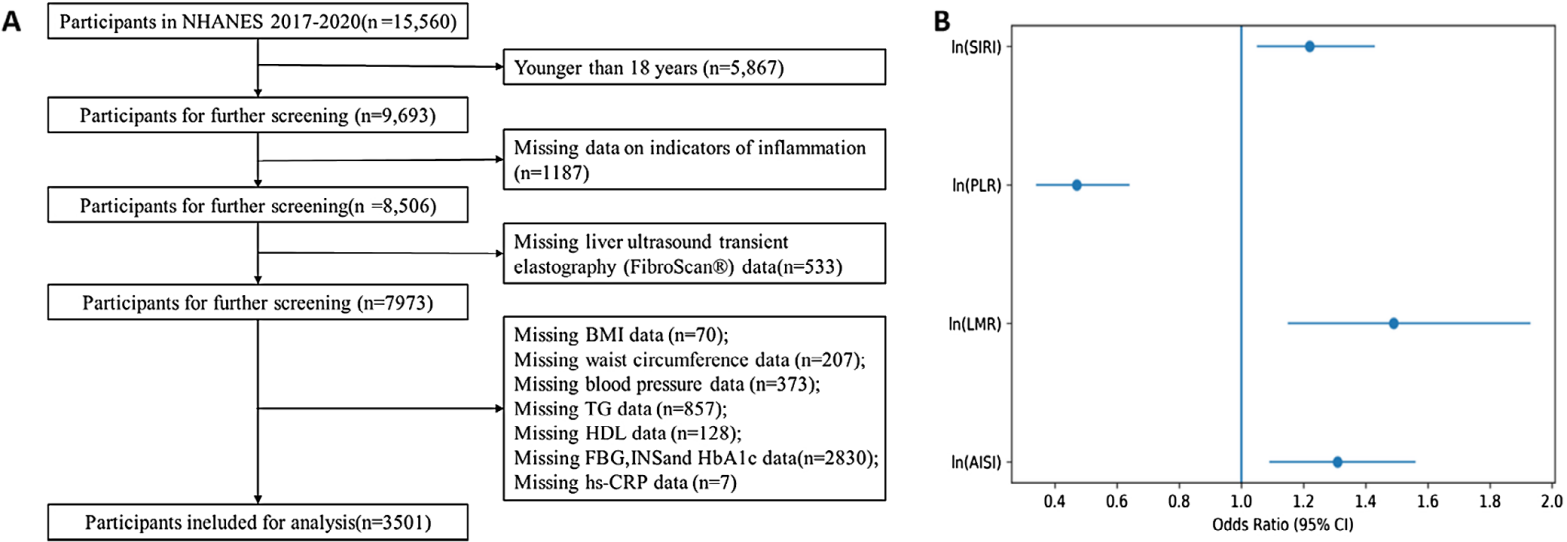
Study flowchart and multivariable‐adjusted associations between inflammatory indices and MAFLD. (A) Flowchart illustrating participant selection from NHANES 2017–2020. (B) Forest plot displaying adjusted odds ratios (Model 3) with 95% confidence intervals for the associations between log‐transformed inflammatory indices and MAFLD.

### 2.2 Definition of MAFLD

MAFLD was diagnosed in the presence of HS identified by biopsy, imaging or blood‐based markers, together with at least one of the following: overweight/obesity, metabolic dysregulation or Type 2 diabetes mellitus (T2DM) [1, 11]. The presence of ≥ 2 criteria defined metabolic dysregulation: (i) waist circumference ≥ 102 cm in men or ≥ 88 cm in women; (ii) blood pressure ≥ 130/85 mmHg or antihypertensive therapy; (iii) triglycerides ≥ 1.70 mmol/L or lipid‐lowering treatment; (iv) HDL cholesterol < 1.0 mmol/L in men or < 1.3 mmol/L in women; (v) prediabetes (fasting glucose 5.6–6.9 mmol/L, 2‐h glucose 7.8–11.0 mmol/L, or HbA1c 5.7%–6.4%); (vi) HOMA‐IR ≥ 2.5; or (vii) hs‐CRP > 2 mg/L.

HS and LF were measured using controlled attenuation parameter (CAP) and liver stiffness measurement (LSM) obtained by transient elastography (FibroScan), a validated noninvasive method [12]. Examinations were conducted at the Mobile Examination Centre after ≥ 3 h of fasting, with ≥ 10 valid measurements required. HS was defined as CAP ≥ 248 dB/m [13] and LF as LSM ≥ 6.4 kPa, based on thresholds validated in Western populations [14].

### 2.3 Inflammatory Indices

The study participants abstained from food for a minimum of 9 h before venipuncture. The complete blood count (CBC) components were measured at the Mobile Examination Center (MEC) using the Beckman Coulter DxH 800 analyzer for white blood cells (WBC), red blood cells (RBC) and platelet counts. The differential WBCs included neutrophils, lymphocytes and monocytes. The CBC components were used to calculate the inflammatory indexes AISI, NLR, LMR, PLR, SII and SIRI, which have been well recognized as predictive indexes for various diseases [12, 13]. The NHANES program has been rigorous in terms of quality control measures for data collection, including standardized procedures, equipment calibration, training of personnel and extensive monitoring of samples to ensure analytical reliability. The formulas for the inflammatory indices were as follows:

$$\text{AISI}=\left(\text{Neutrophils}\times \text{Monocytes}\times \text{Platelets}\right)/\text{Lymphocytes};$$

LMR=Lymphocytes/Monocytes;NLR=Neutrophils/Lymphocytes;

PLR=Platelets/Lymphocytes;

SII=Platelets×Neutrophils/Lymphocytes;

SIRI=Monocytes×Neutrophils/Lymphocytes.

Since the distribution of inflammatory indicators is obviously right‐skewed, natural logarithm conversion is carried out before statistical analysis to reduce skewness, minimize the impact of extreme values and improve the stability of the model. All the inflammation indicators were log‐transformed before being evaluated.

### 2.4 Covariates

Covariate data were collected from laboratory tests, standardized questionnaires and physical examinations. The covariates were chosen based on previous literature and clinical relevance to reduce the possibility of residual confounding. The covariates included age, sex, race/ethnicity, marital status, educational level, smoking status, poverty income ratio (PIR: < 1.30, 1.30–3.50 and > 3.50) [15], alcohol consumption, physical activity (PA), diet and alanine aminotransferase/aspartate aminotransferase (ALT/AST) ratio [16]. The ALT/AST ratio was adjusted for in the model to reflect underlying liver pathology. The potential for the ALT/AST ratio to be an intermediate outcome was recognized to reduce overadjustment bias.

Smoking status was categorized by serum cotinine level: high exposure ≥ 3.0 ng/mL, moderate exposure 0.015–3.0 ng/mL and low exposure < 0.015 ng/mL. Those who consumed ≥ 12 alcoholic drinks in the past year were defined as drinkers [17]. Physical activity was defined as ≥ 600 and < 600 MET minutes per week as adherence and nonadherence to physical activity recommendations, respectively [18].

Considering the impact of nutrition on MAFLD development and progression, nutritional parameters were considered as confounding variables. The total daily energy expenditure (in kcal/day), based on interviews using a 24 h dietary recall method, was incorporated in the fully adjusted model to control for confounding effects related to eating habits. The covariate data are presented in the procedures for the NHANES.

### 2.5 Statistical Assessment

Statistical analysis was carried out in R software (version 4.4.2), with a two‐tailed *p*‐value < 0.05 considered significant. Continuous variables were displayed as mean ± standard deviation or median (IQR) and analyzed via Student's *t*‐test or Mann–Whitney U test, while categorical variables were analyzed using chi‐squared tests. Multiple imputation was used for missing data (< 20%) and variance inflation factor was used to define multicollinearity. Logistic regression models were created to assess the relationship between log‐transformed inflammatory measurements and the odds of developing MAFLD and MAFLD with LF, controlling for factors that could potentially confound this relationship. The use of restricted cubic splines was then utilized to provide dose‐dependent effects, while ROC curves were generated to evaluate the diagnostic performance of these associations and comparison ROC curves were produced. Random forest models were used to evaluate the relative importance of the variables, 500 trees (nTree = 500), three variables are randomly selected for each split (mtry = 3) and the minimum terminal node size is 1 observation (nodesize = 1). The importance of variables was evaluated using the mean decrease Gini index. Subgroup analysis and fully adjusted models were performed according to the presence or absence of diabetes. orest plots were constructed to present adjusted odds ratios (ORs) and 95% confidence intervals (CIs). All analyses used NHANES sampling weights, strata and cluster in the analysis.

## 3 Results

### 3.1 Clinical and Inflammatory Profiles of Participants

After excluding participants with incomplete essential data (Figure 1A), our study sample comprised 3501 participants aged 18 years and older. Table 1 presents the fundamental clinical, metabolic and inflammatory features of the individuals. In comparison to patients without MAFLD, individuals with MAFLD had significantly distinct clinical and metabolic characteristics.

**TABLE 1:** Baseline clinical characteristics of the included individuals.

Variables	Total, *n* = 3501	Non‐MAFLD, *n* = 1531	MAFLD, *n* = 1970	*p*‐value
	*n* (%) or M (Q1‐Q3)	*n* (%) or M (Q1‐Q3)	*n* (%) or M (Q1‐Q3)	
Gender				0.004
Female	1749 (49.78%)	814 (54.71%)	935 (45.80%)	
Male	1752 (50.22%)	717 (45.29%)	1035 (54.20%)	
Age (years)	47.00 (32.00, 61.00)	39.00 (27.00, 56.00)	53.00 (38.00, 64.00)	< 0.001
Race				0.082
Hispanic	801 (15.95%)	294 (14.53%)	507 (17.09%)	
Non‐Hispanic	2700 (84.05%)	1237 (85.47%)	1463 (82.91%)	
Education				0.007
Less than high school	635 (10.48%)	244 (9.04%)	391 (11.63%)	
High school or equivalent or above	2866 (89.52%)	1287 (90.96%)	1579 (88.37%)	
Marital status				< 0.001
Married/cohabitant	2022 (60.63%)	799 (53.18%)	1223 (66.66%)	
Widowed/divorced/separated	741 (18.58%)	301 (18.29%)	440 (18.81%)	
Never married	738 (20.79%)	431 (28.53%)	307 (14.53%)	
PIR				0.083
< 1.30	966 (19.04%)	433 (17.34%)	533 (20.41%)	
≥ 1.30, < 3.50	1386 (34.02%)	575 (32.33%)	811 (35.38%)	
≥ 3.50	1149 (46.94%)	523 (50.33%)	626 (44.20%)	
Drinking status				0.600
Nondrinker	320 (6.58%)	137 (6.20%)	183 (6.88%)	
Drinker	3181 (93.42%)	1394 (93.80%)	1787 (93.12%)	
Smoking status				0.400
Low level	1159 (38.04%)	469 (36.57%)	690 (39.22%)	
Moderate level	1392 (36.99%)	591 (36.62%)	801 (37.29%)	
High level	950 (24.97%)	471 (26.81%)	479 (23.49%)	
Total PA (MET‐minutes/week)				0.001
< 600	1231 (29.74%)	488 (26.13%)	743 (32.66%)	
≥ 600	2270 (70.26%)	1043 (73.87%)	1227 (67.34%)	
BMI (kg/m^2^)	28.30 (24.50, 33.00)	24.60 (22.20, 27.90)	31.20 (27.80, 36.20)	< 0.001
WC (cm)	98.50 (87.30, 110.50)	87.10 (79.90, 95.90)	106.30 (98.10, 117.80)	< 0.001
TC (mg/dL)	181.00 (157.00, 209.00)	177.00 (153.00, 204.00)	185.00 (161.00, 213.00)	0.011
TG (mg/dL)	88.00 (61.00, 134.00)	67.00 (50.00, 96.00)	112.00 (78.00, 160.00)	< 0.001
LDL (mg/dL)	107.00 (85.00, 130.00)	101.00 (83.00, 126.00)	111.00 (88.00, 134.00)	0.009
HDL (mg/dL)	51.00 (42.00, 62.00)	57.00 (48.00, 68.00)	47.00 (40.00, 56.00)	< 0.001
FBG (mmol/L)	5.66 (5.33, 6.16)	5.44 (5.16, 5.77)	5.94 (5.55, 6.61)	< 0.001
HOMA‐IR	2.43 (1.46, 4.26)	1.59 (1.05, 2.43)	3.51 (2.22, 5.95)	< 0.001
Diabetes				< 0.001
No	2740 (84.49%)	1401 (95.15%)	1339 (75.88%)	
Yes	761 (15.51%)	130 (4.85%)	631 (24.12%)	
ALT(U/L)	18.00 (13.00, 26.00)	15.00 (12.00, 21.00)	21.00 (15.00, 31.00)	< 0.001
AST(U/L)	19.00 (16.00, 24.00)	19.00 (16.00, 22.00)	20.00 (16.00, 25.00)	< 0.001
ALT/AST	0.95 (0.76, 1.19)	0.83 (0.71, 1.00)	1.06 (0.87, 1.31)	< 0.001
AISI	233.36 (155.65, 361.20)	207.58 (140.37, 323.00)	263.08 (168.96, 381.09)	< 0.001
NLR	1.93 (1.44, 2.58)	1.86 (1.40, 2.45)	1.96 (1.48, 2.64)	0.015
LMR	3.75 (3.00, 4.67)	3.75 (3.00, 4.75)	3.80 (2.88, 4.60)	0.300
PLR	122.50 (97.89, 150.43)	126.15 (101.36, 158.10)	119.13 (94.12, 146.90)	0.001
SII	446.00 (325.31, 628.09)	424.50 (314.55, 605.53)	464.29 (337.90, 637.96)	0.004
SIRI	1.00 (0.68, 1.47)	0.89 (0.63, 1.33)	1.07 (0.74, 1.59)	< 0.001

*Note:* Medians with interquartile ranges were used to represent non‐normally distributed variables and counts and percentages were used to represent categorical variables. Baseline characteristics were compared between individuals with and without MAFLD using the Mann–Whitney U test for non‐normally distributed continuous variables and the Chi‐squared test for categorical variables. *p*‐values reflected whether observed differences between the two groups were statistically significant.

Abbreviations: ALT, alanine aminotransferase; AST, aspartate aminotransferase; BMI, body mass index; FBG, fasting blood glucose; HDL, high‐density lipoprotein; HOMA‐IR, homeostatic model assessment of insulin resistance; LDL, low‐density lipoprotein cholesterol; PA, physical activity; PIR, family poverty income ratio; TC, total cholesterol; TG, triglycerides; WC, waist circumference.

Individuals with MAFLD were older (median 53 vs. 39 years, *p* < 0.001), more often married (66.66% vs. 53.18%, *p* < 0.001), and had substantially higher body mass index (31.20 vs. 24.60 kg/m^2^, *p* < 0.001) and waist circumference (106.30 vs. 87.10 cm, *p* < 0.001). Physical activity levels were significantly lower in the MAFLD group (*p* = 0.001).

Regarding systemic inflammation, all indices differed significantly between groups except LMR (*p* = 0.300). NLR, AISI, SII and SIRI were higher in individuals with MAFLD, whereas PLR was considerably lower. Overall, MAFLD was characterized by pronounced metabolic dysfunction accompanied by heightened systemic inflammation.

### 3.2 Associations Between Inflammatory Markers and MAFLD

Multivariable logistic regression analysis was performed to investigate the relationship between log‐transformed inflammatory indices and MAFLD. In the unadjusted model (Model 1), ln (AISI), ln (NLR), ln (SII), and ln (SIRI) were positively associated with MAFLD, whereas ln (PLR) showed an inverse association. Most of these relationships persisted after adjusting for demographic data (Model 2).

In the fully adjusted Model 3, controlling for demographic, socioeconomic, lifestyle and metabolic covariates, several indices remained significantly associated with MAFLD. Each one‐unit increase in ln (AISI) was associated with 31% higher odds of MAFLD (OR = 1.31; 95% CI: 1.09–1.56; *p* = 0.007) (Table 2). Similarly, ln (LMR) and ln (SIRI) were associated with 49% (OR = 1.49; 95% CI: 1.15–1.93; *p* = 0.006) and 22% (OR = 1.22; 95% CI: 1.05–1.43; *p* = 0.016) greater odds, respectively. In contrast, the relationship between ln (PLR) was inverse, with each increasing unit corresponding to a 53% decrease in the odds of MAFLD (OR = 0.47; 95% CI: 0.34 to 0.64; *p* < 0.001). Tertile analysis showed clear dose–response effects. Participants who had the highest tertiles of both ln (AISI) and ln (SIRI) exhibited substantially elevated chances of MAFLD relative to those who had the minimum tertiles of the same predictors. The relationship for ln (PLR) was graded but inverse (all *p* for trend < 0.05). Overall, both AISI and SIRI were consistently positive predictors of MAFLD, while PLR was a robust negative predictor.

**TABLE 2:** Associations of inflammatory markers, analyzed as continuous variables and tertiles, with the odds of MAFLD.

Participants(ln‐transformed)	Model 1		Model 2		Model 3	
	OR (95% CI)	*p*‐value	OR (95% CI)	*p*‐value	OR (95% CI)	*p*‐value
ln (AISI)						
Continuous	1.54 (1.34–1.77)	< 0.001	1.44 (1.23–1.68)	< 0.001	1.31 (1.09–1.56)	0.007
T1	ref.		ref.		ref.	
T2	1.35 (1.03–1.76)	0.029	1.30 (0.96–1.74)	0.081	1.12 (0.80–1.57)	0.473
T3	2.04 (1.53–2.71)	< 0.001	1.77 (1.32–2.36)	0.001	1.45 (1.04–2.04)	0.033
p for trend	< 0.001		< 0.001		0.027	
ln (NLR)						
Continuous	1.28 (1.02–1.62)	0.036	0.97 (0.76–1.23)	0.78	0.91 (0.74–1.12)	0.333
T1	ref.		ref.		ref.	
T2	1.09 (0.87–1.38)	0.44	1.05 (0.82–1.36)	0.657	0.95 (0.71–1.26)	0.667
T3	1.45 (1.15–1.84)	0.003	1.15 (0.90–1.47)	0.254	1.05 (0.85–1.31)	0.613
p for trend	0.002		0.5		0.8	
ln (LMR)						
Continuous	0.92 (0.76–1.11)	0.36	1.61 (1.24–2.08)	0.001	1.49 (1.15–1.93)	0.006
T1	ref.		ref.		ref.	
T2	1.09 (0.85–1.41)	0.474	1.50 (1.20–1.89)	0.002	1.40 (1.10–1.80)	0.012
T3	0.88 (0.73–1.07)	0.185	1.40 (1.12–1.75)	0.005	1.37 (1.07–1.75)	0.018
p for trend	0.2		< 0.001		0.002	
ln (PLR)						
Continuous	0.55 (0.41–0.74)	< 0.001	0.44 (0.34–0.59)	< 0.001	0.47 (0.34–0.64)	< 0.001
T1	ref.		ref.		ref.	
T2	0.92 (0.70–1.23)	0.57	0.90 (0.69–1.18)	0.426	0.85 (0.62–1.17)	0.285
T3	0.73 (0.55–0.95)	0.022	0.62 (0.49–0.79)	0.001	0.64 (0.49–0.84)	0.005
p for trend	0.024		< 0.001		< 0.001	
ln (SII)						
Continuous	1.34 (1.08–1.67)	0.01	1.25 (0.99–1.59)	0.059	1.11 (0.88–1.41)	0.333
T1	ref.		ref.		ref.	
T2	1.19 (0.99–1.44)	0.064	1.17 (0.95–1.44)	0.129	1.03 (0.81–1.32)	0.764
T3	1.53 (1.18–1.99)	0.003	1.43 (1.09–1.86)	0.012	1.21 (0.91–1.61)	0.164
p for trend	0.001		0.009		0.3	
ln (SIRI)						
Continuous	1.59 (1.40–1.80)	< 0.001	1.32 (1.14–1.52)	0.001	1.22 (1.05–1.43)	0.016
T1	ref.		ref.		ref.	
T2	1.45 (1.13–1.85)	0.005	1.39 (1.07–1.80)	0.016	1.24 (0.93–1.66)	0.133
T3	1.97 (1.61–2.42)	< 0.001	1.56 (1.27–1.92)	< 0.001	1.39 (1.09–1.76)	0.012
p for trend	< 0.001		< 0.001		0.006	

*Note:* Model 1 was the crude model; Model 2 was adjusted for education level, race, gender, PIR, as well as age; Model 3 was adjusted for race, PIR, drinking status, gender, education level, smoking status, age, PA, as well as ALT/AST.

#### 3.2.1 Subgroup Analysis by Diabetes Status

Table 3 demonstrates that the associations between inflammatory indices and MAFLD were predominantly uniform among participants, regardless of diabetes status. In both subgroups, a higher ln (AISI) was significantly linked to a greater likelihood of MAFLD, with a more pronounced effect noted in participants with diabetes (OR≈1.42) relative to those without diabetes (OR≈1.25). In the same way, ln (SIRI) stayed positively linked and ln (PLR) stayed negatively linked in both groups. Notably, ln (PLR) exhibited a more robust protective association in the diabetes subgroup (OR≈0.41) relative to the non‐diabetes subgroup (OR≈0.52). ln (LMR) exhibited a positive association in both groups, whereas ln (NLR) and ln (SII) did not demonstrate a significant association with MAFLD. Overall, these findings indicate that inflammatory indices are consistently associated with MAFLD across diabetes status, with slightly stronger effects observed among individuals with diabetes.

**TABLE 3:** Subgroup analysis of associations between inflammatory indices and MAFLD stratified by diabetes status.

Variables (ln‐transformed)	Diabetes (Yes) OR (95% CI)	*p*‐value	Diabetes (No) OR (95% CI)	*p*‐value
ln (AISI)	1.42 (1.12–1.80)	0.004	1.25 (1.01–1.54)	0.041
ln (NLR)	1.18 (0.88–1.59)	0.261	0.89 (0.70–1.13)	0.335
ln (LMR)	1.62 (1.18–2.23)	0.003	1.39 (1.05–1.84)	0.021
ln (PLR)	0.41 (0.28–0.60)	< 0.001	0.52 (0.38–0.71)	< 0.001
ln (SII)	1.19 (0.89–1.58)	0.231	1.08 (0.84–1.39)	0.547
ln (SIRI)	1.31 (1.06–1.63)	0.014	1.19 (1.01–1.41)	0.038

#### 3.2.2 Visualization of Associations

Forest plots were created to graphically illustrate the modified relationships between inflammatory indicators and MAFLD (Figure 1B). In accordance with the regression findings, ln (AISI), ln (LMR), and ln (SIRI) exhibited a positive association with MAFLD, but ln (PLR) had a significant negative association.

### 3.3 Associations Between Inflammatory Markers and MAFLD With LF

Multivariable logistic regression analyses were conducted to examine the association among log‐transformed systemic inflammatory indices and LF among patients with MAFLD (Table 4). When not adjusted for any confounding variables, the prevalence of LF increased with increasing levels of ln (AISI), ln (NLR), and ln (SIRI) but decreased with increasing levels of ln (PLR). These relationships remained significant after adjusting for all the aforementioned variables sequentially.

**TABLE 4:** Associations between systemic inflammation indices (as continuous variables) and the odds of MAFLD with LF.

Participants (ln‐transformed)	Model 1		Model 2		Model 3	
	OR (95% CI)	*p*‐value	OR (95% CI)	*p*‐value	OR (95% CI)	*p*‐value
ln (AISI)	1.31 (1.05–1.63)	0.019	1.27 (1.01–1.58)	0.041	1.27 (1.00–1.61)	0.049
ln (NLR)	1.49 (1.11–2.00)	0.01	1.41 (1.02–1.94)	0.039	1.42 (1.01–1.99)	0.046
ln (LMR)	0.91 (0.65–1.28)	0.567	0.98 (0.65–1.47)	0.914	0.95 (0.60–1.49)	0.804
ln (PLR)	0.68 (0.51–0.90)	0.008	0.64 (0.51–0.81)	0.001	0.65 (0.51–0.84)	0.004
ln (SII)	1.28 (0.98–1.68)	0.07	1.23 (0.95–1.60)	0.111	1.23 (0.93–1.63)	0.128
ln (SIRI)	1.46 (1.17–1.82)	0.002	1.42 (1.09–1.84)	0.013	1.43 (1.08–1.88)	0.017

In the comprehensively adjusted Model 3, where the prevalence of LF was further adjusted for the effects of total energy intake (kcal/day) to control for the effects of dietary factors, a one‐unit increase in ln(AISI) was associated with a 27% elevated prevalence of LF (OR = 1.27; 95% CI = 1.00–1.61; *p* = 0.049). ln (NLR) and ln (SIRI) associated to 42% (OR = 1.42; 95% CI = 1.01–1.99; *p* = 0.046) and 43% higher prevalence of LF (OR = 1.43; 95% CI = 1.08–1.88; *p* = 0.017), respectively. Conversely, ln (PLR) was associated with a 35% lower prevalence of LF (OR = 0.65; 95% CI = 0.51–0.84; *p* = 0.004). No substantial association was seen between ln (LMR) or ln (SII) and the probability of LF.

Importantly, further adjustment for dietary intake resulted in minimal changes to the effect estimates. Overall, increased systemic inflammatory markers were found to be associated with a greater chance of fibrosis in patients with MAFLD, whereas a greater PLR was inversely associated with fibrosis.

### 3.4 Dose–Response Associations Between Inflammatory Indices and Odds of MAFLD and MAFLD With LF

The RCS analysis was used to determine the presence of a nonlinear association between the log‐transformed levels of inflammatory indices and the odds of MAFLD and MAFLD with LF (Figure 2A–J). The association between ln (AISI), ln (NLR), ln (LMR), ln (PLR), ln (SII) and ln (SIRI) and MAFLD was found to be linear (P for nonlinearity > 0.05). The prevalence of MAFLD was found to be increased with increased levels of ln (AISI), ln (SII) and ln (SIRI). Conversely, an inverse association was observed for ln (PLR). The association between the above‐mentioned inflammatory indices and MAFLD with LF was also found to be linear for ln (AISI), ln (NLR), ln (PLR) and ln (SIRI); hence, their predictive value was confirmed (Figure 2G–J).

**FIGURE 2:**
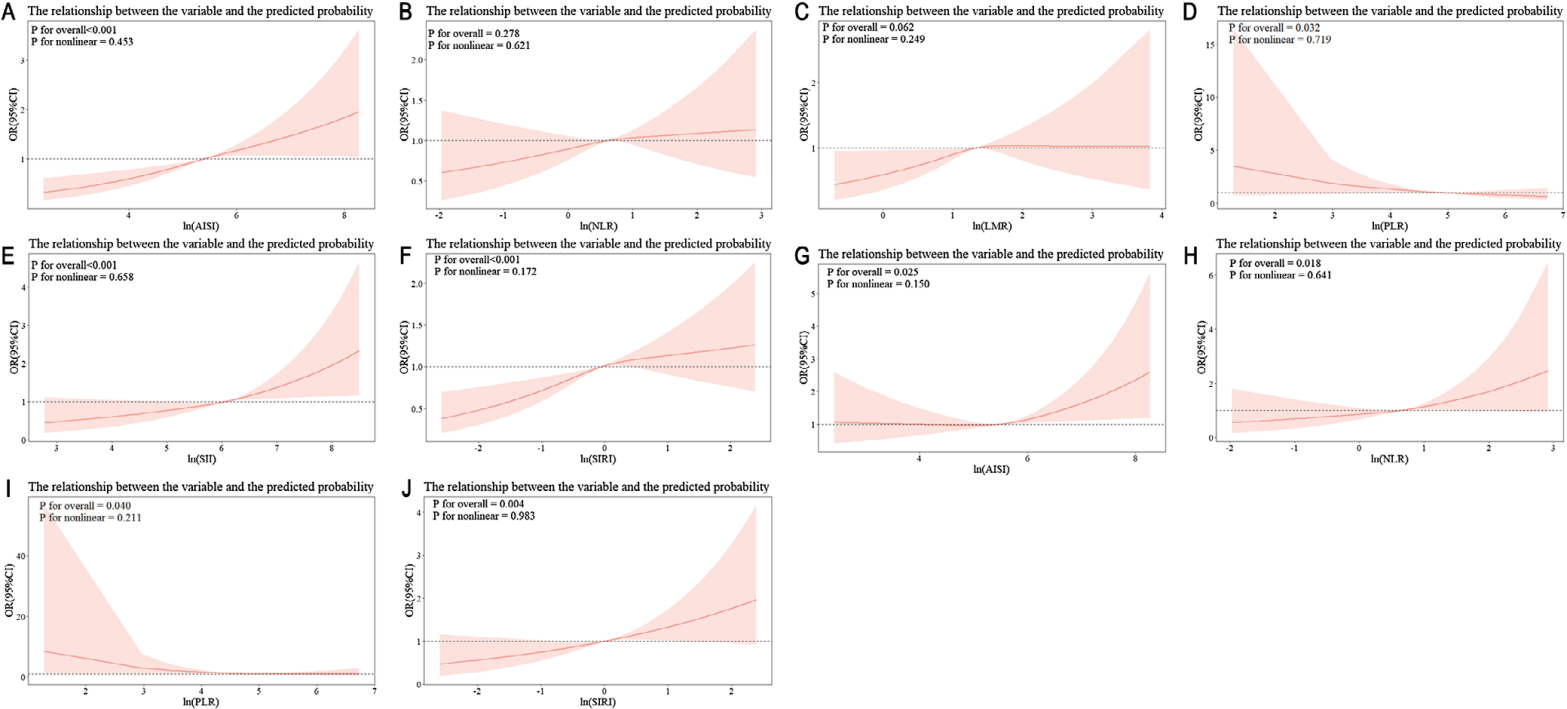
RCS analyses showing the associations between MAFLD and (A) ln (AISI), (B) ln (NLR), (C) ln (LMR), (D) ln (PLR), (E) ln (SII), and (F) ln (SIRI); RCS analyses showing the associations between MAFLD with LF and (G) ln (AISI), (H) ln (NLR), (I) ln (PLR), and (J) ln (SIRI).

### 3.5 Performance of Inflammatory Markers and Their Components for Assessing the Odds of MAFLD and MAFLD With LF

ROC analysis was conducted to assess the diagnostic efficacy of systemic inflammatory indicators for MAFLD (Table 5 and Figure 3A). Among the log‐transformed indices, ln (AISI) exhibited the greatest discriminative capability (AUC = 0.752; 95% CI: 0.736–0.769), followed by ln (SIRI), ln (SII), ln (PLR), ln (LMR), and ln (NLR), which indicated similar but somewhat inferior performance (AUC range: 0.746–0.750).

**TABLE 5:** Performance of inflammation markers for assessing the odds of MAFLD.

Variables (ln‐transformed)	AUC	95% CI	Sensitivity	Specificity	Threshold
ln (AISI)	0.752	0.736,0.769	77.11%	61.27%	0.523
ln (NLR)	0.746	0.730,0.763	71.07%	65.97%	0.575
ln (LMR)	0.747	0.731,0.763	67.61%	69.69%	0.599
ln (PLR)	0.747	0.731,0.763	74.92%	61.92%	0.539
ln (SII)	0.749	0.733,0.765	76.55%	60.81%	0.534
ln (SIRI)	0.750	0.734,0.766	76.29%	62.12%	0.531

**FIGURE 3:**
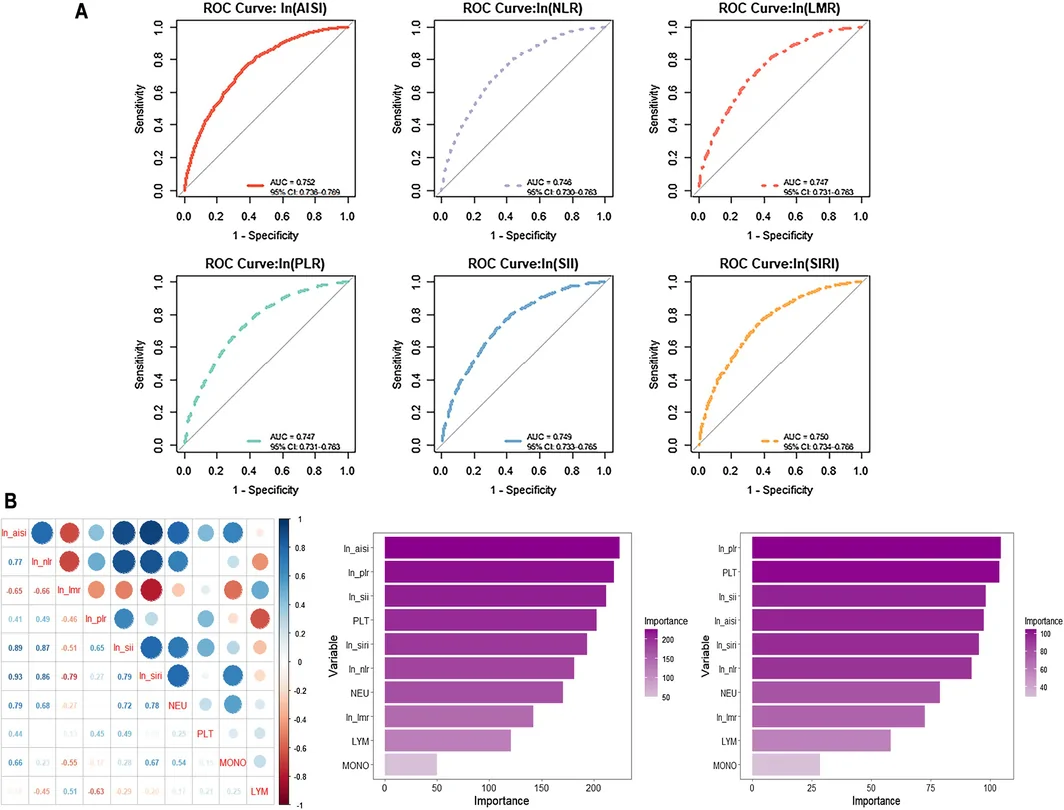
Diagnostic performance and importance of inflammatory indices for predicting MAFLD and liver fibrosis. (A) Comparative receiver operating characteristic (ROC) curves illustrating the discriminative performance of ln (AISI), ln (NLR), ln (LMR), ln (PLR), ln (SII), and ln (SIRI) for predicting MAFLD. (B) Spearman's association matrix showing relationships among inflammatory indices and their hematological components. (C) Variable importance ranking of inflammatory markers and their components for identifying MAFLD based on random forest (RF) analysis. (D) Variable importance ranking of inflammatory markers and their components for identifying MAFLD with liver fibrosis (LF) based on RF analysis.

Comparative ROC curves demonstrated comparable diagnostic efficacy across indices, with ln (AISI) exhibiting slightly enhanced predictive value. Association analysis demonstrated interconnections among inflammatory indicators and their constituents (Figure 3A). Random forest analysis further validated ln (AISI) as the paramount predictor of MAFLD, while ln (PLR) emerged as the most significant predictor of MAFLD‐related liver fibrosis (Figure 3B–D).

## 4 Discussion

The associations between inflammatory markers like LMR, PLR, AISI, SII, NLR and SIRI and the likelihood of MAFLD and MAFLD with LF were examined in a nationally representative sample of adults from the United States. After adjusting for all confounding variables by using the logarithmic method, increased levels of AISI and SIRI were found to be significantly linked to increased odds of MAFLD, while no significant association was found between ln (SII), ln (NLR), ln (LMR) and the prevalence of MAFLD. A decreased level of PLR was also found to be linked to increased MAFLD. In the case of MAFLD with LF, increased levels of AISI, NLR and SIRI showed strong positive associations, whereas PLR showed a continued inverse association. ln (LMR) and ln (SII) were not significantly associated with MAFLD in the context of LF. The results of the ROC analysis revealed that the AISI performed nominally best among a set of indices with very similar and moderate discriminative ability. The RF analysis revealed that PLR has a good association with MAFLD with LF. The RCS analysis revealed a dose–response relationship for the association between the AISI and MAFLD as well as the PLR and MAFLD with LF. Overall, these results indicate that these inflammatory indices may be used as a tool for the prediction of MAFLD.

The AISI, defined as a composite inflammatory index that encompasses the interactions between neutrophils, platelets, monocytes and lymphocytes based on the results of CBC tests, provides a holistic approach to the assessment of the inflammatory status [19]. In recent times, the clinical significance of the AISI has been established in various conditions. Sanchez‐Bao et al. [20] established a significant relationship between the onset of Type 2 diabetes mellitus and the value of the AISI. Kim et al. [21] established a relationship between the levels of the AISI and depression. The elevated value of the AISI has been identified as a specific marker for cardiovascular mortality in several individuals according to Chen et al. [22]. However, the relationship between the value of the AISI and MAFLD is still in the early stages of research.

In addition, in the current study, it was found that ln (AISI) had the strongest discriminatory ability in predicting MAFLD (AUC: 0.752), compared with other inflammatory markers. This was confirmed by the results of the RF model, where AISI had a better performance compared with other markers under investigation. Increasing one unit in ln (AISI) (Taking logarithm e≈2.718, the original value AISI increases by 2.718 times) is associated with a 31% rise in the odds of having MAFLD, as indicated by a trend in tertiles (P for trend < 0.05).

In addition, a linear dose–response connection was found among ln (AISI) and the probability of having MAFLD, as indicated by a *p* value for nonlinearity of 0.453 in RCS analysis, adjusting for other variables. In addition, a significant association was found between ln (AISI) and the odds of having LF among individuals with MAFLD, shown by an OR of 1.27, 95% CI: 1.00–1.61, *p* = 0.049.

The improved efficacy of AISI can be viewed as a result of the synergistic effects of immune cells that enhance hepatic inflammation and fibrogenesis. Neutrophils and monocytes release reactive oxygen species, proteolytic enzymes and pro‐inflammatory cytokines that lead to hepatocyte injury and the activation of hepatic stellate cells, an important mechanism in fibrosis. Platelets also enhance inflammation by releasing transforming growth factor β and platelet‐derived growth factor, thereby aiding the deposition of the extracellular matrix. Conversely, lymphocytes have immunomodulatory effects and lower lymphocyte levels may be indicative of poor immune regulation and increased inflammation. By combining all the immune cells, AISI probably reflects the accumulated inflammation that occurs during the progression of MAFLD.

This pro‐inflammatory environment is associated with the onset and advancement of MAFLD with LF through mechanisms like insulin resistance, hepatic fat accumulation, and oxidative stress [23]. The current research offers new insight into the pathophysiology of MAFLD. The results also indicate that AISI may serve as an efficient tool for the early detection of MAFLD as a potential biomarker. Numerous studies have shown the association between the development and progression of NAFLD and the levels of PLR, MLR, and NLR. However, the results were not consistent. Yang et al. [24] found that NAFLD patients had a high NLR and LMR as well as a low PLR compared to healthy subjects. The authors found that NLR is associated with the risk of NAFLD but is not associated with the progression of NAFLD. Liu et al. [25] found that NLR and LMR were linearly and positively associated with the risk of MAFLD. However, the association among PLR and the risk of MAFLD is U‐shaped. A negative relationship has been found between PLR and NAFLD. A U‐shaped relationship has been found between PLR and the progression of LF [26]. Wang et al. [27] did not find a clear dose–response relationship between LMR, NLR, PLR, and MASLD. The results may be due to the difference in the diagnostic criteria for NAFLD, MAFLD, and MASLD. The authors found that the spectrum of the above diseases is similar [28].

In the multivariable analysis, after adjusting for all the confounding factors, NLR did not show any association with MAFLD. However, it did show a significant linear relationship with MAFLD‐related LF. This suggests that the effect of neutrophil‐induced inflammation might be more significant in the later stages of the progression of the disease [29].

There was no significant association between ln (LMR) and MAFLD in the multivariable logistic regression or restricted cubic spline analyses. Similarly, ln (LMR) was not significantly associated with MAFLD‐related liver fibrosis.

The literature shows that the values of PLR are lower in patients with NAFLD compared with healthy controls. Another study has confirmed the negative relationship between PLR values and NAFLD [30]. The PLR index highlights the complex role of platelet regulation of the immune response in the progression of MAFLD [24]. Rather than showing a protective effect against the progression of the disease, a low PLR may indicate an imbalance in the regulation of the immune response. This imbalance may result from the inability of the lymphocytes to exert an effective immune response.

Moreover, the RF model revealed that PLR is superior to other atherosclerotic factors in the prediction of MAFLD‐related LF, implying that PLR can be a potential biomarker for clinical assessment. The study also aimed to investigate the relationship between MAFLD and cumulative inflammatory markers (SII and SIRI). There was no significant correlation between ln (SII) and the prevalence of MAFLD and LF. Previous studies on the relationship between these factors have shown mixed results. Yuan et al. [31] observed an inverted U‐shaped relationship between SII and HS. There was no significant relationship between LF and SII. Mirchandani et al. [32] observed a U‐shaped relationship between SII and NAFLD. Another study by Wang et al. [33] observed a linear positive association between SII, SIRI and MASLD risk.

In the current research, the systemic immune‐inflammation index did not retain its statistical significance after full adjustment, reinforcing the importance of considering metabolic and dietary factors. Conversely, an increased level of the systemic immune‐inflammatory response index displayed a linear dose–response relationship with increased probability of MAFLD as well as MAFLD with LF, suggesting that inflammatory indexes that include monocyte‐related inflammation may be better markers of chronic inflammatory states.

The “multiple‐hit hypothesis” has replaced the “two‐hit hypothesis” as an explanation for the pathogenesis of MAFLD. This is because the “multiple‐hit hypothesis” is more comprehensive, considering the synergistic impact of metabolic disorders, disorders of the gut microbiome, inflammatory responses, as well as genetic factors [34, 35, 36]. Excessive inflammatory responses are strongly associated with irreversible hepatic damage, fibrosis, and carcinogenesis.

Platelets, neutrophils, monocytes, and lymphocytes, based on CBCs, are dynamic markers of inflammatory burden. The responses mediated by neutrophils and monocytes are associated with liver damage; platelets are involved in fibrogenesis, and abnormal lymphocytes are responsible for perpetuating chronic inflammation [37]. The prognostic value of these inflammatory markers based on these immune cells has been established in various diseases [38]. Most importantly, the addition of dietary covariates resulted in minimal changes in effect estimates, thereby validating the observed associations and reducing concerns of dietary confounding [39].

The robustness and clinical significance of the identified relationships are strengthened by the consistency of results across subgroup and machine learning studies. The results' potential therapeutic relevance is supported and their credibility is increased when multiple analytical methods agree. This concordance across multiple analytical approaches enhances the reliability of the findings and supports their potential clinical applicability.

Using a large nationally representative population from the NHANES study cohort, the current study provides generalizable evidence. Inflammation biomarkers, being noninvasive and inexpensive measures that can be easily reproduced, make them suitable for routine screening. The current study represents one of the first nationally representative head‐to‐head comparisons of these six inflammation‐derived indices and demonstrates that the AISI is a predictor of MAFLD prevalence and that PLR is a predictor of fibrosis prevalence.

By using simple and routine hematological indices such as AISI and PLR, MAFLD and hepatic fibrosis risk can be stratified early on from a clinical perspective due to their low cost of use. The value of these indices is enhanced in settings where advanced imaging technology or other more invasive diagnostic tests may not be readily available; therefore, they can help in making decisions regarding patient management strategies in primary care practices as well as in areas with limited resources.

There are some limitations to the study, which need to be acknowledged. Firstly, the study design of NHANES, being a cross‐sectional study, does not allow the establishment of a cause‐and‐effect relationship. Additionally, reverse causation cannot be ruled out. Although inflammatory markers may contribute to the progression of MAFLD, they may also be a consequence of underlying metabolic and hepatic pathophysiology. Therefore, the findings need to be interpreted cautiously. Further studies are needed to ascertain the relationship. Some other limitations are the absence of histological confirmation and the possibility of residual confounding despite extensive control. However, if the findings are verified by further studies, the inflammatory markers may be useful in the early identification of high‐risk patients.

## 5 Conclusion

In conclusion, this study has established that there are significant associations between blood cell‐derived inflammatory indices and both MAFLD and liver fibrosis in a nationwide sample of individuals. The AISI was identified as a predictive index for MAFLD, whereas the PLR performed best at predicting the prevalence of developing fibrosis. These results suggest that blood cell‐derived inflammatory indices may be useful to develop non‐invasive, cost‐effective means of identifying patients early and using an objective measure for risk stratification of MAFLD.

## Author Contributions

Hao Chen and Qinmei Chen contributed to the conceptualization and study design. Hao Chen performed the methodology, formal analysis, and investigation. Xiangqian Wang contributed to conceptualization, formal analysis, and investigation. Yixian He contributed to formal analysis and investigation. Hao Chen and Qinmei Chen prepared the original draft of the manuscript. Qinmei Chen and Xiqiao Zhou contributed to the review and editing. Xiqiao Zhou provided resources, funding acquisition, and supervision. All authors, Hao Chen, Qinmei Chen, Xiangqian Wang, Yixian He, and Xiqiao Zhou, reviewed and approved the final version of the manuscript and agreed to be accountable for all aspects of the work.

## Funding

A project funded by the Priority Academic Program Development of Jiangsu Higher Education Institutions.

## Disclosure

No AI tools were used in the design, conduct, analysis and writing of this study.

## Ethics Statement

Ethics review and approval were waived for this study because it used publicly available, de‐identified data from the National Health and Nutrition Examination Survey (NHANES). Because NHANES data are anonymized and collected with informed consent by the National Center for Health Statistics, additional ethical approval was not required for this secondary data analysis.

## Conflicts of Interest

The authors declare no conflicts of interest.

## Data Availability

The data used in this study were obtained from the publicly available National Health and Nutrition Examination Survey (NHANES) 2017–2020 database, maintained by the U.S. National Center for Health Statistics (NCHS). The datasets analyzed in the present study are publicly accessible at the NHANES repository: https://www.cdc.gov/nchs/nhanes/. No new datasets were generated during the current study. The data analyzed in this study can be accessed and downloaded from the NHANES database in accordance with its public‐use data policy.
